# Socioeconomic position, perceived weight, lifestyle risk, and multimorbidity in young adults aged 18 to 35 years: a Multi-country Study

**DOI:** 10.1186/s12889-023-16234-1

**Published:** 2023-07-15

**Authors:** Ashleigh Craig, Asanda Mtintsilana, Witness Mapanga, Siphiwe N. Dlamini, Shane A. Norris

**Affiliations:** 1 SAMRC/Wits Developmental Pathways for Health Research Unit, Faculty of Health Sciences, University of the Witwatersrand, Johannesburg, South Africa; 2 DSI-NRF Centre of Excellence in Human Development, Faculty of Health Sciences, University of Witwatersrand, Johannesburg, South Africa; 3 Noncommunicable Disease Research Division, Wits Health Consortium (PTY) Ltd, Johannesburg, South Africa; 4 School of Physiology, Faculty of Health Sciences, University of Witwatersrand, Johannesburg, South Africa; 5 School of Human Development and Health, Global Health Research Institute, University of Southampton, Southampton, UK

**Keywords:** Multimorbidity, Weight perceptions, Lifestyle risk, Socioeconomic position, Multi-country

## Abstract

**Background:**

Multimorbidity-risk is established early in life, therefore reducing modifiable risk factors such as overweight or obesity may, in part, tackle the burden of multimorbidity in later life.

**Methods:**

We made use of a cross-sectional online survey that included young adults (18-35yrs old) from three countries – Kenya, South Africa, and the United Kingdom (*n* = 3000). Information pertaining to socio-demographic, health, lifestyle, and perceived weight was collected. Additionally, the sum of affirmed morbidities was used to determine a morbidity score. Likewise, a lifestyle risk score was calculated based on information obtained from questions surrounding four unhealthy lifestyle behaviours, namely current smoking, alcohol consumption, physical inactivity, and overweight/obese weight status as a confirmed clinic condition. We further explored differences in socioeconomic position, and the prevalence of perceived weight, multimorbidity, and lifestyle risk factors between the three countries. We also determined the odds ratio of multimorbidity with perceived weight as a main predictor variable. We furthermore performed a generalised structural equation model to determine whether the association between socioeconomic position and multimorbidity was mediated via perceived weight and/or lifestyle risk.

**Results:**

Socioeconomic position, weight perceptions, lifestyle risk, and multimorbidity varied significantly across the different economic countries. Higher morbidity (by > 11.9%) and lifestyle risk (by > 20.7%) scores were observed in those who reported an overweight weight perception when compared to those with an underweight or normal weight perception. In pooled analyses, the odds ratio in developing 2 or more morbidities increased multiple times in those who perceived themselves as overweight (all models: OR ≥ 2.241 [95% CI ≥ 1.693; ≥ 2.966] *p* < 0.001), showing a larger odds ratio with high significance in those who reported 3 or more morbidities (all models: OR ≥ 3.656 [95% CI ≥ 2.528; ≥ 5.286] *p* < 0.001). Furthermore, this study showed that an overweight weight perception partially mediated (*p* ≤ 0.001) the association between socioeconomic position and multimorbidity.

**Conclusions:**

This study confirmed poorer health outcomes in those who perceived themselves as overweight. The findings from this study further emphasise the importance of targeted intervention strategies directed at raising weight-related awareness and potentiating risk factors, specifically in those who reside in lower economic developed countries.

**Supplementary Information:**

The online version contains supplementary material available at 10.1186/s12889-023-16234-1.

## Background

The prevalence of obesity among young adults in both high-income countries (HICs) and low- to middle-income countries (LMICs) has reached epidemic proportions [1], which further leads to an increase in multimorbidity [2]. Multimorbidity is defined as the co-occurrence of multiple health conditions [3], and has been associated with poorer health outcomes and the increased use of health- and social-care services with associated costs [4]. The prevalence of multimorbidity in the general population is high, but even higher in obese when compared to non-obese individuals [5]. Over 27% of young adults from the United Kingdom (UK) are obese, with corresponding numbers in other countries across Sub-Saharan Africa being even higher [6]. As multimorbidity-risk is established early in life, reducing modifiable risk factors like unfavourable lifestyle behaviours [7], unhealthy dietary intake [8], and physical inactivity [9] in young adulthood in essence, proves among the best efforts to reduce the burden of multimorbidity in later life. Generally, the outcome of individuals with multimorbidity is worse across Sub-Saharan Africa in comparison to HICs, which could be attributed to lack of access to healthcare services, lengthy time of diagnosis and the overall management of confirmed aliments [10]. Those who reside in LMICs typically have low multimorbidity awareness and may erroneously consider chronic conditions to be an unavoidable consequence of the ageing process [10].

Lifestyle modifications leading to modest weight loss has shown to prevent cardiovascular disease [11]. However, changing health-related behaviour remains challenging as the effectiveness of intervention and preventive strategies seem difficult to sustain in the long-term [12]. This may in part, be related to an individual’s perception of overweight and obesity [2]. For instance, individuals of any size who perceive themselves to be of “normal” weight may underestimate the importance of routine health checks and further fail to appreciate the need to improve their dietary and physical activity habits [13].

Notably, due to several factors, namely epigenetic, physiologic, and socioeconomic, health conditions may not translate directly from HICs to Sub-Saharan African settings. All these factors exemplify the significance of generating Sub-Saharan Africa-specific multimorbidity evidence – including trends and drivers in multimorbidity, effective prevention, and intervention strategies. Moreover, no consensus has been reached on whether perceptions of being overweight or underweight foster or discourage healthy lifestyle behaviours and whether these perceptions relate to adverse health outcomes. Therefore, this study aimed to explore weight perceptions, lifestyle risk, and multimorbidity among young adults (aged 18-35yrs old) from Kenya (upper low-income), South Africa (upper middle-income), and the UK (high-income).

## Methods

**Fig. 1:**
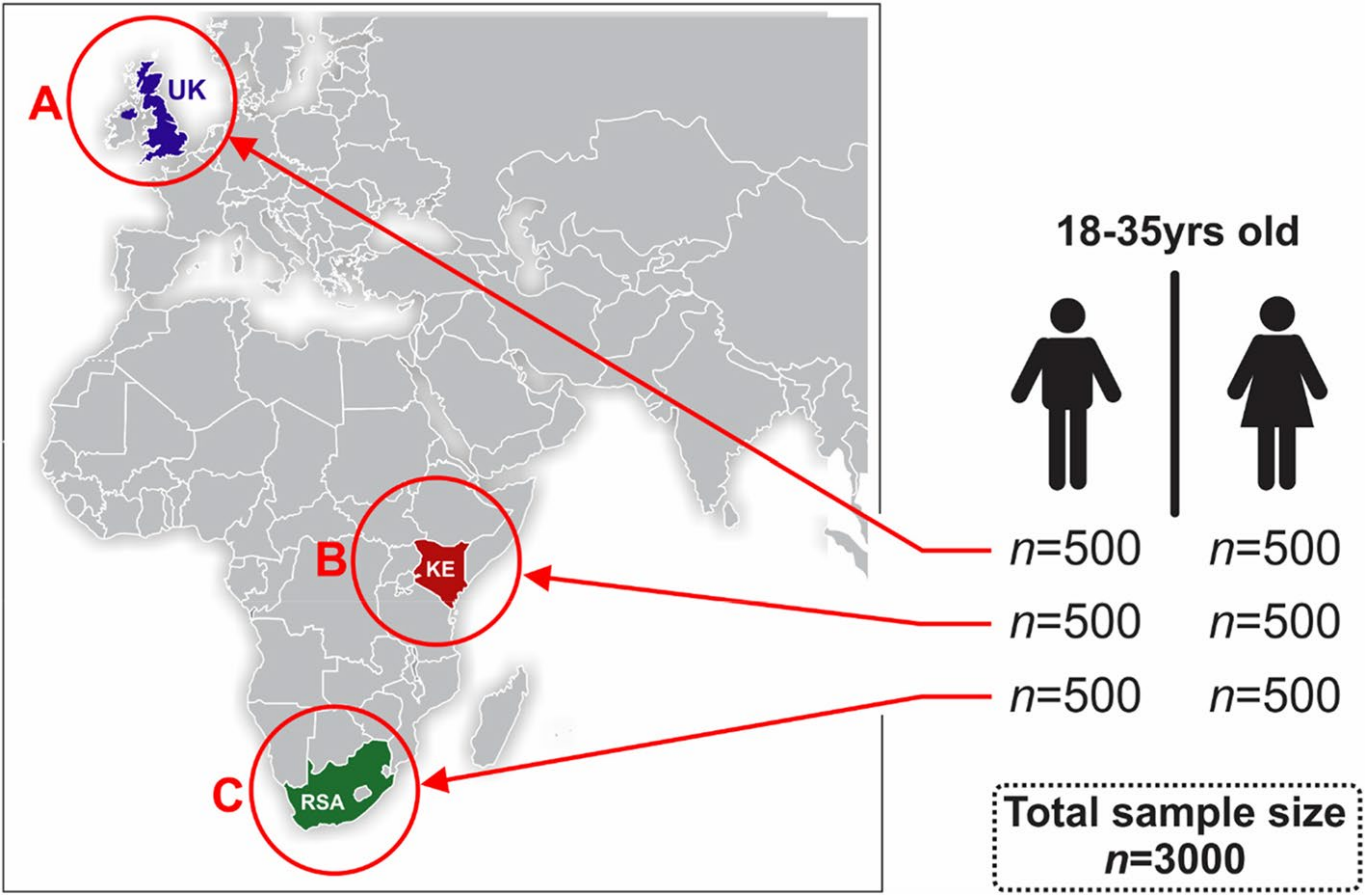
Geographic location outlining sample distribution: A) United Kingdom (UK), B) Kenya (KE) and C) South Africa (RSA)

This cross-sectional study made use of survey data collected in April 2022 from three countries – Kenya, South Africa, and the UK. The survey was concluded when 1000 respondents from each country completed the survey and were deemed valid through the backend checks described below. Therefore, the study sample (*n* = 3000) was not randomly recruited but targeted and included young adults (*n* = 1000; 18–35 years old) from each country with an equal sex distribution as outlined in Fig. 1.

### Survey integrity and processes

**Fig. 2:**
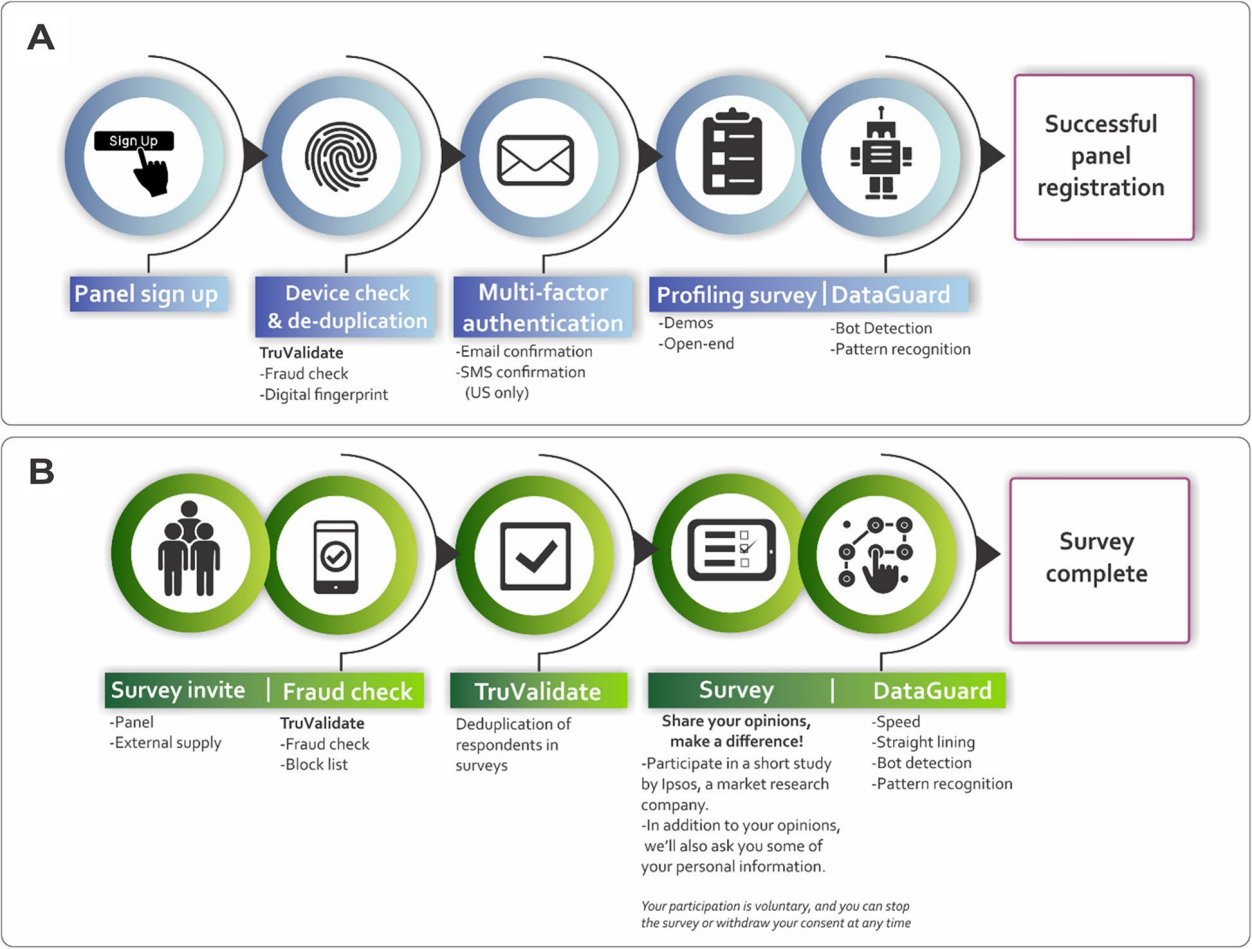
Ipsos i-Say respondent registration process and survey completion **A**) panel registration and **B**) in-survey completion

The survey questionnaire was distributed electronically through Ipsos proprietary i-Say panel as outlined in Fig. 2, namely in the form of 2 processes A) panel registration and B) in-survey completion. The Ipsos protocol has been published elsewhere [14], however, in brief, prospective Ipsos panellists interested in partaking in the survey – were recruited via a multi-source recruitment process referred by various partner panels known to meet the quality of Ipsos and Global Research Standards. Ipsos additionally partners with TransUnion – an American consumer credit reporting agency – for the use of digital fingerprinting technology known as TruValidate. TruValidate passively collects respondent information associated with the device entering the panel registration and survey link. A unique identifier was issued to the respondents’ device based on its distinctive characteristics. The device was then reviewed against a set of business rules and regulations created by Ipsos to score the likelihood of fraud (fraud check). The device identification is checked to prevent the creation of multiple panel accounts and to ensure that multiple entries into the same survey are ultimately avoided (deduplication). The TruValidate process is deployed at the panel registration and then again in-survey. It is still, however, possible for fraudulent respondents to create multiple digital fingerprints by using different device and account combinations to bypass systems. Digital fingerprinting is one element within a multi-layered suite of tools and systems to mitigate this limitation. Others include the use of Multi-Factor authentication during recruitment (respondent email account and/or cellphone number confirmation), the checking of respondents’ contact details against existing i-Say accounts and, checking blocked accounts. Lastly, Ipsos incorporates a newly rebuilt and rebranded tool known as Dataguard, which is a cyber robot designed to detect open ends (copied and pasted text, programmatic text insertion, look-up versus known bad, or suspect responses; exaggerated, and/or unrealistic typing speed; exaggerated, or unrealistic reaction time after question loads), data pattern recognition (algorithms to review new panellists and flag those that match the profile of known fraudsters); speeding (measures the pace of survey completion in answers per minute) and straight lining (identifies respondents who gave the same answer to all statements in a grid question). In the event a respondent is caught by Dataguard, respondents are automatically removed from the survey in real-time.

Through verified data audits of those respondents who participated in each country, in combination with multiple checks and linked to remuneration, only one response is possible from each respondent. Furthermore, the recruitment was based on the individual level with an exclusion criterion that no two respondents could participate from the same household. Respondents were recruited to be representative of the specific country's population across age and gender groups that had access to the internet. This may, however, not necessarily be representative of the entire youth or general population of Kenya, South Africa, and the UK.

### Survey data collected

Information pertaining to the respondent (age and sex), socio-demographic determinants (household assets – included a tally of all major operational household amenities (e.g., refrigerator, washing machine, television, computer etc.)), health-related information (morbidities and lifestyle behavioural risk factors), and weight perceptions (British Survey Questionnaire [15]) were collected via the survey. In this cross-sectional study, household asset score was used as an indicator of affluence and referred to as socioeconomic position (SEP) throughout this study. This score was based on standard measures used in the Demographic and Health Surveys household questionnaire (www.measuredhs.com).

To assess perceived weight, respondents were asked a series of binary questions pertaining to their weight. Questions included, “Do you think of yourself as underweight?”, “Do you think of yourself as about the right weight?” and “Do you think of yourself as overweight?”. From respondents’ affirmative responses (yes), we were able to determine the number of respondents who perceived themselves as either underweight, normal weight, or overweight.

To assess overall health, respondents were asked a series of questions pertaining to existing conditions that had been diagnosed by a doctor or healthcare professional (i.e., hypertension, myocardial infarction, hypercholesterolemia/hyperlipidemia, obesity, HIV/AIDS, tuberculosis, lung disease, mental health risk, stroke, diabetes, asthma, cancer, liver disease, chronic kidney disease, and/or joint disease). Seeing that multimorbidity is commonly understood to be the coexistence of multiple health conditions an individual experiences at one time, multimorbidity for each respondent was calculated based on the number of existing known clinic conditions the respondent answered they had experienced (yes = 1; no = 0) (i.e., a condition count). The multimorbidity score was further categorised into 3 groups based on those respondents who reported null or 1 condition (0–1 morbidity); those with comorbidity (i.e., more than 1 condition) (2 morbidities); and those with multimorbidity (i.e., more than 2 conditions) (≥ 3 morbidities), respectively. Due to the lack of respondents that reported no clinic conditions across all three countries, respondents who reported null or 1 condition were grouped together (i.e., 0–1 morbidities).

Additionally, a lifestyle risk score was calculated based on information obtained from questions surrounding four unhealthy lifestyle behaviours, namely current smoking, alcohol consumption, physical inactivity, and overweight/obese weight status as a known condition. Smoking and alcohol consumption were defined from a binary question and confirmed with an affirmative (yes) response. To assess physical activity, respondents were asked a series of questions pertaining to time/week spent engaging in either vigorous or moderate physical activity to which a moderate-to-vigorous physical activity (MVPA) variable was computed. MVPA was calculated by adding all the time spent in moderate and vigorous physical activity per week. Physical inactivity was therefore defined as a MVPA < 150 min/week as recommended by the World Health Organisation [16]. Notably, respondents who reported implausible physical activity domains (min/day) were excluded from the statistical analyses according to the Global Physical Activity Questionnaire (GPAQ) data cleaning [17]. Lastly, overweight/obesity was confirmed with an affirmative (yes) response to having the known condition from a list of health-related questions. For each of the four selected lifestyle risk factors, respondents received a score of 1 if they practised unhealthy behaviour, otherwise received a score of 0. A total lifestyle risk score ranged from 0 to 4, indicating the sum of these four scores. Higher scores indicate an unhealthier lifestyle.

### Statistical analyses

For all statistical analyses, IBM® SPSS® version 28 (IBM Corporation, Armonk, New York), Stata® version 17.0 (StataCorp, College Station, TX, USA) and GraphPad Prism version 5.03 for Microsoft® Windows (GraphPad Software, San Diego, California, USA) were used to analyse and plot the data. Variables were tested for normality using the Kolmogorov–Smirnov test and QQ-plots. For group comparisons, analyses of variance (ANOVA) were used. Proportions across both continuous and categorical variables were determined with crosstabs with significant differences indicated by Chi-square tests and presented as percentages. For multimorbidity as an outcome, respondents were stratified according to the number of known conditions/morbidities they experienced (i.e., group 1: 0–1 morbidities, group 2: 2 morbidities or group 3: 3 or more morbidities). Multivariable adjusted multinomial logistic regressions were performed to determine the odds of having multimorbidity with weight perceptions and socio-demographic determinants (age, sex, SEP, and country) as predictor and confounder variables, respectively.

Additionally, a generalised structural equation model (gSEM) was used to test the relationship between SEP and multimorbidity, and whether this relationship was mediated by perceived weight, lifestyle risk score (as a latent variable) and/or confirmed overweight/obesity variables. Direct (unmediated), indirect (mediated) and total effects were computed and recorded, and the proportion of the total effect mediated was calculated. Modifications to pathways and adding/removing variables were made iteratively and the Akaike and Bayesian Information Criteria (IC) of each model were compared. The final model was selected for having a low IC and high theoretical relevance. Direct, indirect, and total effects were calculated using non-linear combination estimates. Due to the nature of the gSEM, only respondents that presented with a complete dataset of all variables of interest (i.e., SEP, perceived weight, multimorbidity, lifestyle risk and overweight/obesity) were included in gSEM analyses (*n* = 1626). Hence respondents with any missing variables of interest were excluded from the gSEM analyses (*n* = 1374). Additionally, the gSEM model was conceptualised ahead of any data analyses.

## Results

Due to the nature of the survey which targets 1000 participants completing the survey per country, a total of 3000 respondents (50.0% female) participated in the survey. Thus, no data was missing. However, post-survey data cleaning removed 1374 respondents from the gSEM analyses as per GPAQ data cleaning guidelines [17]. In Kenya, respondents were predominantly adults between 20-25yrs (34.2%), while respondents in the UK and South Africa were largely adults aged 30-35yrs (≥ 29%). Additionally, the largest proportion of respondents in the UK were those who reported a marital status of married or co-habiting (57.1%), while a large proportion of respondents from both South Africa and Kenya reported being single (≥ 55%). Furthermore, the SEP was recorded in the range of 13.5 to 14.5, with the highest mean SEP score (14.5), surprisingly reported in South Africa (SD < 2.3).

### Prevalence of socioeconomic position, multimorbidity outcomes, lifestyle risk, and weight perceptions

**Fig. 3:**
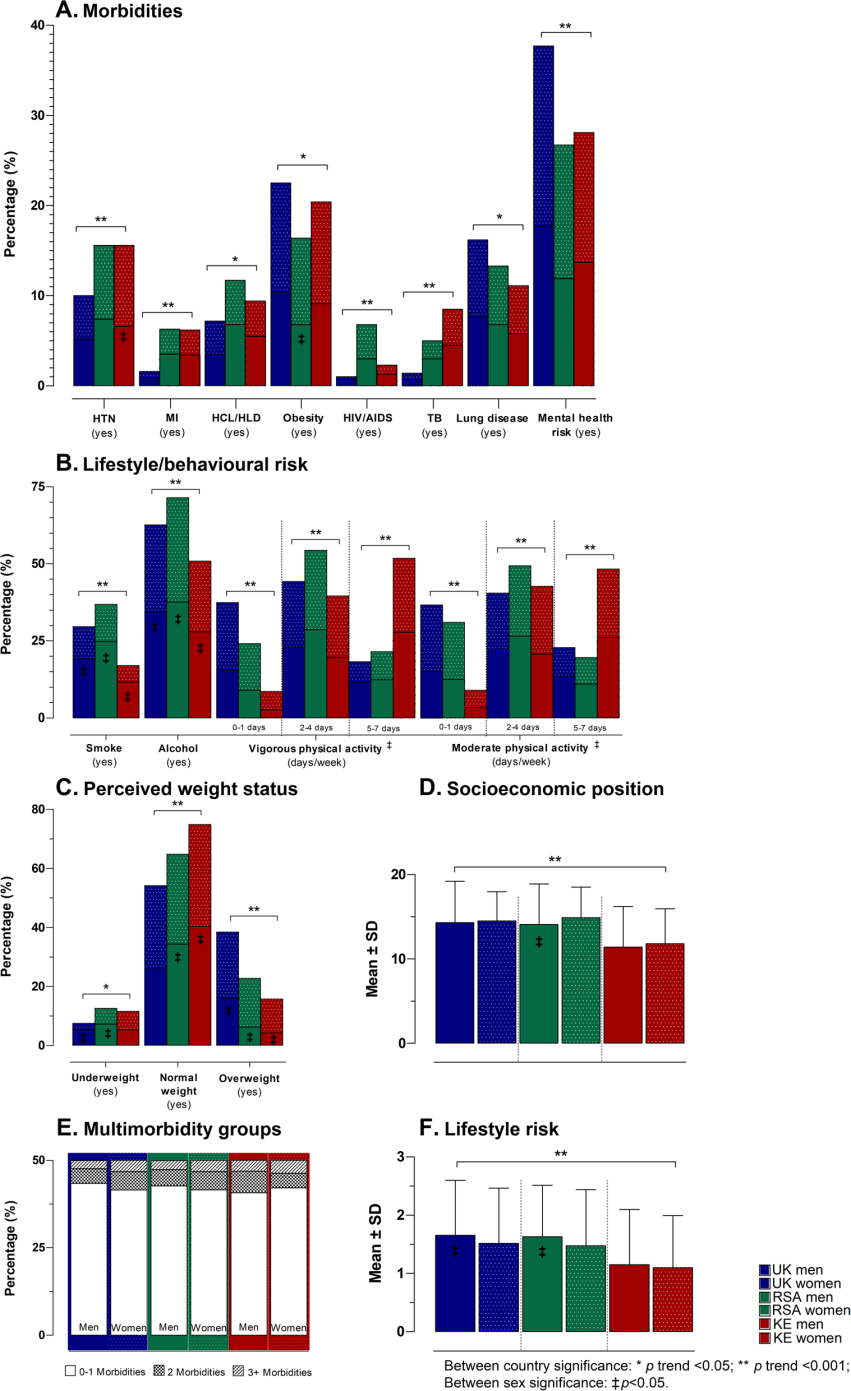
Characteristics of the study population stratified by **A** morbidities; **B** lifestyle/behavioural risk; **C** perceived weight status, **D** socioeconomic position, **E** multimorbidity groups, and **F** lifestyle risk. Abbreviations: UK – United Kingdom; RSA – Republic of South Africa; KE – Kenya; SD - standard deviation; HTN - hypertension; MI - myocardial infarction; HCL/HLD - hypercholesterolemia/hyperlipidemia; HIV/AIDS - human immunodeficiency virus/acquired immunodeficiency syndrome; TB - tuberculosis

Although the SEP of South Africa and the UK were comparable, respondents from both countries presented with a significantly higher number of resources than Kenyan respondents (*p* < 0.001). The overall sum of morbidities (cumulative morbidities experienced) did not differ between the countries (*p* = 0.91) (Supplementary Table S1). Introspectively, several morbidities significantly differed across the countries and between sexes (all *p* < 0.001) (Fig. 3; Supplementary Table S2), I.e., the prevalence of hypercholesterolemia/hyperlipidaemia (11.7%) and HIV/AIDS (6.8%) was reported higher in South Africa when compared to the two other countries. Lung disease (16.2%), obesity (22.5%) and mental health risk (37.8%) were reported higher in the UK when compared to South Africa and Kenya. Additionally, the prevalence of hypertension and myocardial infarction, although comparable between South Africa and Kenya (15.6% and ≤ 6.3% respectively), were significantly higher in these two countries when compared to the UK. With regards to lifestyle risk factors, South Africa reported the highest prevalence of smoking (36.9%) and alcohol consumption (71.5%) yet, reported the most physically active (63.7%) respondents (MVPA > 60 min/day). Comparing the lifestyle risk score across the countries showed that both the UK and South Africa had significantly higher scores when compared to Kenya (both mean scores ≥ 1.55). More specially, men from both the UK and South Africa had higher lifestyle risk scores when compared to their female counterparts (both *p* ≤ 0.021).

When comparing those stratified in the weight perception groups (Table 1), the overall sum of morbidities significantly differed across the weight perception groups, with the highest mean morbidity score (1.42), reported in the overweight perception group (SD = 0.85). Within the overweight perception group, self-reported morbidities such as obesity (49.6%) and asthma/lung disease (15.7%) were significantly higher in this group, while mental health risk (29.4%) and joint disease (10.0%) reported lower in this group.

**Table 1:** Health profiling and lifestyle risk factors of the study population stratified by weight perceptions

		**Underweight** (*n* = 265)	**Normal weight** (*n* = 1608)	**Overweight** (*n* = 637)	***p-*trend**
**Health profile**					
Morbidity score	(mean ± SD)	1.25 ± 0.72^c^	1.22 ± 0.88^b^	1.42 ± 0.85^bc^	** < 0.001**
Hypertension (yes)	*n* (%)	28 (10.6%)	211 (13.1%)	93 (14.6%)	0.26
Myocardial infarction (yes)	*n* (%)	11 (4.2%)	88 (5.5%)^b^	10 (1.6)^b^	** < 0.001**
Stroke (yes)	*n* (%)	4 (1.5%)	21 (1.3%)	4 (0.6%)	0.34
Hypercholesterolemia / hyperlipidaemia (yes)	*n* (%)	16 (6.0%)	147 (9.1%)	55 (8.6%)	0.25
Diabetes (yes)	*n* (%)	15 (5.7%)	130 (8.1%)	40 (6.3%)	0.18
Obesity (yes)	*n* (%)	20 (7.5%)^c^	162 (10.1%)^b^	316 (49.6%)^bc^	** < 0.001**
HIV/AIDS (yes)	*n* (%)	14 (5.3%)^c^	52 (3.2%)	13 (2.0%)^c^	**0.038**
Tuberculosis (yes)	*n* (%)	23 (8.7%)^c^	88 (5.5%)^b^	11 (1.7%)^bc^	** < 0.001**
Asthma (yes)	*n* (%)	31 (11.7%)	190 (11.8%)^b^	100 (15.7%)^b^	**0.039**
Cancer (yes)	*n* (%)	2 (0.8%)	20 (1.2%)	4 (0.6%)	0.38
Liver disease (yes)	*n* (%)	5 (1.9%)	17 (1.1%)	5 (0.8%)	0.34
Chronic kidney disease (yes)	*n* (%)	5 (1.9%)	20 (1.2%)	4 (0.6%)	0.23
Mental health risk (anxiety/depression/bi-polar) (yes)	*n* (%)	113 (42.6%)^ac^	498 (31.0%)^a^	187 (29.4%)^c^	** < 0.001**
Joint disease (arthritis) (yes)	*n* (%)	44 (16.6%)^c^	315 (19.6%)^b^	64 (10.0%)^bc^	** < 0.001**
**Lifestyle/behavioural risk**					
Smoke (yes)	*n* (%)	94 (35.5%)^ac^	399 (24.8%)^a^	160 (25.1%)^c^	**0.001**
Alcohol (yes)	*n* (%)	175 (66.0%)	964 (60.0%)	407 (63.9%)	0.065
Physically inactivity (MVPA < 60 min/day)	*n* (%)	95 (35.8%)	439 (27.3%)	285 (44.7%)	** < 0.001**
Lifestyle risk score	(mean ± SD)	1.45 ± 0.949^ac^	1.22 ± 0.894^ab^	1.83 ± 0.967^bc^	** < 0.001**

*Abbreviations*: *n* number of participants. Bold values denote statistical significance (*p* < 0.05)

^**a**^Significant difference between underweight and normal weight

^**b**^significant difference between normal weight and overweight

^**c**^significant difference between underweight and overweight

### Associations of weight perceptions, health outcomes, and socio-demographics

**Fig.4:**
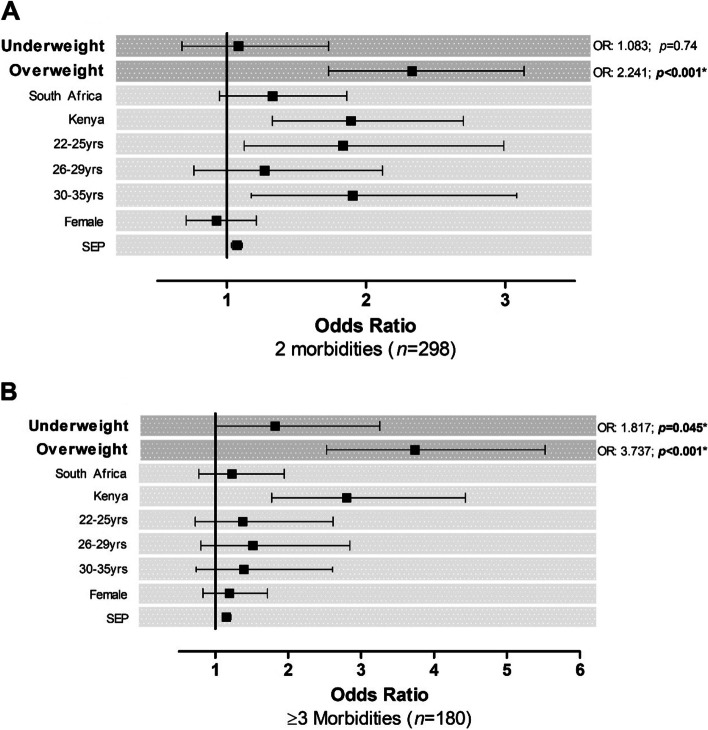
Multivariable adjusted multinomial logistic regressions to determine the odds ratio (OR) of multimorbidity [**A**) 2 morbidities and **B**) ≥ 3 morbidities] with weight perceptions as an independent predictor. Abbreviations: *n* – number of participants; SEP – socioeconomic position. Reference variables in the model include: 0–1 Morbidity, normal weight perception, United Kingdom, 18–25 years of age and male sex. *Bold values denote statistical significance (*p* < 0.05)

We performed univariate and multivariable adjusted multinomial logistic regressions (Supplementary TableS 3 and Fig. 4) to determine the odds of having multimorbidity (determined by having either 2 morbidities or ≥ 3 morbidities) with a varying degree of perceived weight (model 1) and further determined if this relationship is independent of socio-demographic characteristics (model 2: age, sex, and country; model 3: age, sex, country, and SEP). Throughout the models, we determined that, when compared to respondents with a normal weight perception, respondents with an overweight weight perception were multiple times more likely to present with multimorbidity of 2 morbidities compared to their 0–1 morbidity counterparts (model 1: OR, 2.241 [95% CI 1.693; 2.966] *p* < 0.001; model 2: OR, 2.426 [95% CI 1.803; 3.265] *p* < 0.001; model 3: OR, 2.330 [95% CI 1.731; 3.136] *p* < 0.001). Additionally, our results showed that the likelihood of having a harsher degree of multimorbidity with 3 or more morbidities increased by more than 1.5 times with an underweight weight perception (model 1: OR, 1.789 [95% CI 1.009; 3.173] *p* = 0.047; model 2: OR, 1.819 [95% CI 1.021; 3.242] *p* = 0.042; model 3: OR, 1.817 [95% CI 1.014; 3.257] *p* = 0.045) and by more than 3.5 times with an overweight weight perception (model 1: OR, 3.656 [95% CI 2.528; 5.286] *p* < 0.001; model 2: OR, 4.037 [95% CI 2.732; 5.967] *p* < 0.001; model 3: OR, 3.737 [95% CI 2.528; 5.525] *p* < 0.001) with each unit increase in weight perceptions, when compared to those with a normal weight perception. The significance reached in model 1 was confirmed independent of socio-determinants (models 2 and 3).

## Structural model analyses

**Fig.5:**
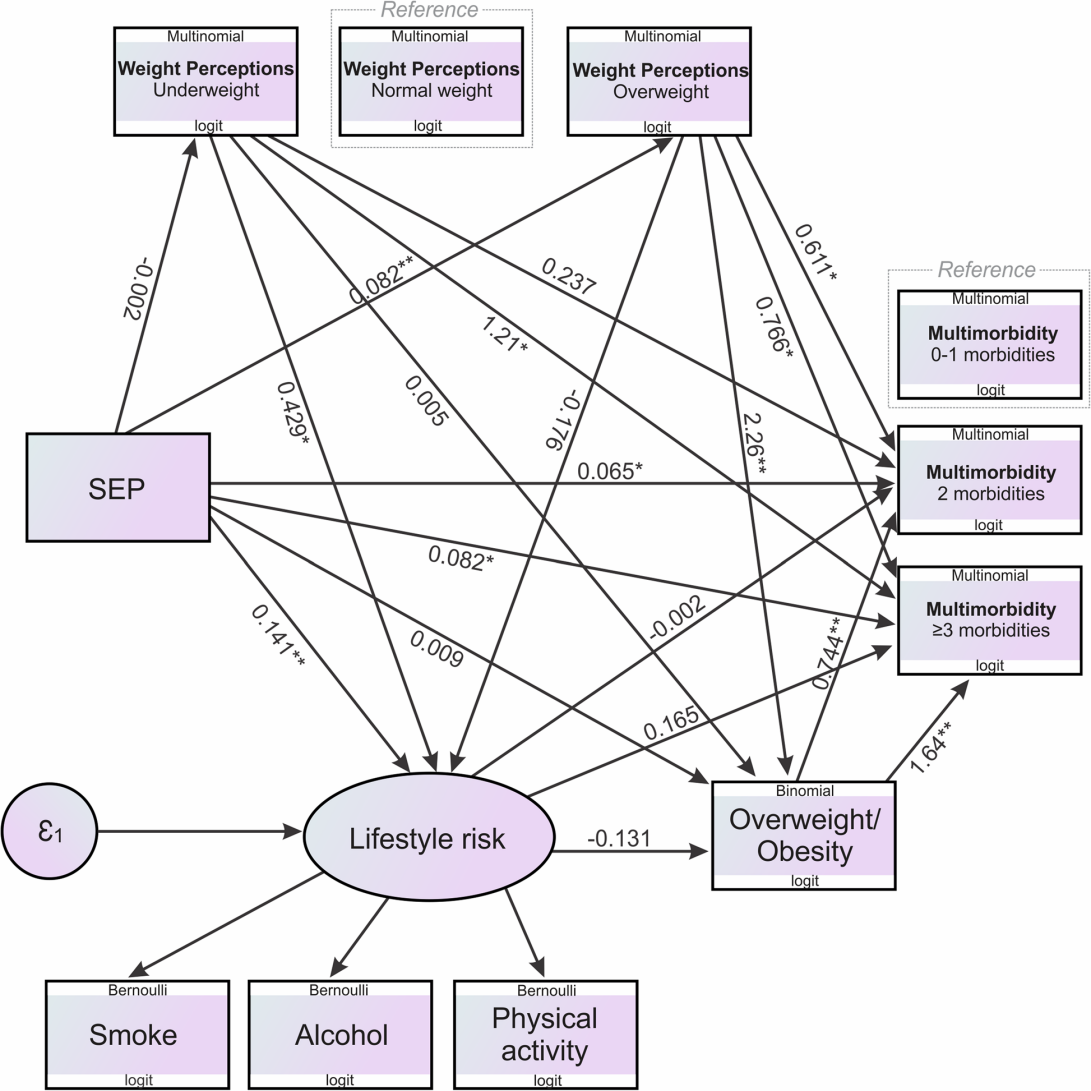
Structural equation model for SEP, weight perceptions, multimorbidity, lifestyle risk and overweight/obesity. Abbreviations: SEP – socioeconomic position. * *p* < 0.05; ** *p* ≤ 0.001

The gSEM was constructed a priori to assess the impact of SEP, weight perceptions, multimorbidity, lifestyle risk and confirmed overweight/obesity (Fig. 5 and Table 2). We found positive and significant total effects of SEP on multimorbidity of both 2 morbidities and ≥ 3 morbidities, lifestyle risk, the overweight weight perception group and having overweight/obesity as a confirmed aliment (p ≤ 0.023). The direct effect of SEP on multimorbidity either through weight perceptions, lifestyle risk or being overweight/obese showed significant effects (p ≤ 0.048). The results revealed a significant indirect effect of SEP on 2 morbidities and ≥ 3 morbidities (p ≤ 0.031), with 43.5% and 42.8% of the indirect effect being mediated via the overweight weight perception group, respectively. These results show that overweight weight perception partially mediates the association between SEP and multimorbidity of 2 or more morbidities. Additionally, significant indirect effect of an overweight weight perception on 2 morbidities and ≥ 3 morbidities (p ≤ 0.001), equated to 72.5% and 95.1% of the indirect effect being mediated by having overweight/obesity as a confirmed aliment, respectively. These results, therefore, further indicate that being overweight/obese additionally partially mediates the association between perceived weight and multimorbidity of 2 or more morbidities.

**Table 2:** Generalised structural equation model in a pooled sample of respondents for socioeconomic position, weight perceptions, multimorbidity, lifestyle risk, and having overweight/obesity as a confirmed condition (*n* = 1626)

**Exposure**	**Outcome**	**Total effect**		**Direct effect**		**Indirect effect**		**Proportion of total effect mediated**
		**Estimate (95% CI)**	***p* value**	**Estimate (95% CI)**	***p* value**	**Estimate (95% CI)**	***p* value**	
**Effect of SEP on multimorbidity via weight perceptions**								
SEP	0–1 Morbidities	Reference	-	Reference	-	Reference	-	Reference
	2 Morbidities via underweight	0.064 (0.011; 0.117)	**0.018**	0.065 (0.012; 0.117)	**0.015**	–0.001 (–0.010; 0.008)	0.90	-
	2 Morbidities via normal weight	Reference	-	Reference	-	Reference	-	Reference
	2 Morbidities via overweight	0.115 (0.053; 0.176)	** < 0.001**	0.065 (0.012; 0.117)	**0.015**	0.050 (0.013; 0.087)	**0.008**	43.5%^†^
	≥ 3 Morbidities via underweight	0.079 (–0.014; 0.173)	0.097	0.082 (0.001; 0.164)	**0.048**	–0.003 (–0.049; 0.043)	0.90	-
	≥ 3 Morbidities via normal weight	Reference	-	Reference	-	Reference	-	Reference
	≥ 3 Morbidities via overweight	0.145 (0.050; 0.239)	**0.003**	0.082 (0.001; 0.164)	**0.048**	0.062 (0.006; 0.120)	**0.031**	42.8%^†^
**Effect of SEP on multimorbidity via lifestyle risk**								
SEP	0–1 Morbidities	Reference	-	Reference	-	Reference	-	Reference
	2 Morbidities via lifestyle risk	0.065 (0.018; 0.111)	**0.007**	0.065 (0.012; 0.117)	**0.015**	0.000 (–0.023; 0.022)	0.98	-
	≥ 3 Morbidities via lifestyle risk	0.105 (0.034; 0.177)	**0.004**	0.082 (0.001; 0.164)	**0.048**	0.023 (–0.011; 0.058)	0.18	-
**Effect of weight perceptions on multimorbidity via lifestyle risk**								
Underweight	0–1 Morbidities	Reference	-	Reference	-	Reference	-	Reference
	2 Morbidities via lifestyle risk	0.237 (–0.350; 0.823)	0.43	0.237 (–0.352; 0.827)	0.43	–0.001 (–0.069; 0.067)	0.98	-
	≥ 3 Morbidities via lifestyle risk	0.611 (0.217; 1.01)	**0.002**	1.21 (0.452; 1.97)	**0.002**	0.000 (–0.027; 0.028)	0.98	-
Overweight	0–1 Morbidities	Reference	-	Reference	-	Reference	-	Reference
	2 Morbidities via lifestyle risk	1.28 (0.529; 2.03)	**0.001**	0.611 (0.217; 1.00)	**0.002**	–0.001 (–0.048; 0.189)	0.24	-
	≥ 3 Morbidities via lifestyle risk	0.737 (1.00; 1.37)	**0.023**	0.766 (0.128; 1.40)	**0.019**	–0.029 (–0.088; 0.030)	0.33	-
**Effect of SEP on multimorbidity via overweight/obesity**								
SEP	0–1 Morbidities	Reference	-	Reference	-	Reference	-	Reference
	2 Morbidities via overweight/obesity	0.072 (0.011; 0.132)	**0.020**	0.065 (0.012; 0.117)	**0.015**	0.007 (–0.024; 0.038)	0.65	-
	≥ 3 Morbidities via overweight/obesity	1.65 (1.06; 2.25)	** < 0.001**	0.082 (0.001; 0.164)	**0.048**	0.015 (–0.005; 0.082)	0.65	-
**Effect of weight perceptions on multimorbidity via overweight/obesity**								
Underweight	0–1 Morbidities	Reference	-	Reference	-	Reference	-	Reference
	2 Morbidities via overweight/obesity	0.070 (–0.510; 0.650)	0.81	0.237 (–0.352; 0.827)	0.43	0.004 (–0.426; 0.434)	0.99	-
	≥ 3 Morbidities via overweight/obesity	1.64 (0.821; 2.47)	** < 0.001**	1.21 (0.452; 1.97)	**0.002**	0.009 (–0.938; 0.956)	0.99	-
Overweight	0–1 Morbidities	Reference	-	Reference	-	Reference	-	Reference
	2 Morbidities via overweight/obesity	2.33 (2.03; 2.62)	** < 0.001**	0.611 (0.217; 1.00)	**0.002**	1.69 (0.744; 2.63)	** < 0.001**	72.5%^†^
	≥ 3 Morbidities via overweight/obesity	3.90 (3.25; 4.56)	** < 0.001**	0.766 (0.128; 1.40)	**0.019**	3.71 (2.30; 5.12)	** < 0.001**	95.1%^†^

*Abbreviation*s: *n* number of participants, *SEP* socioeconomic position

^*^Significant effect, *p* < 0.05, ^†^partial mediation, *p* < 0.05, ^‡^inconsistent mediation, *p* < 0.05

## Discussion

Overweight/obesity has consistently been associated with adverse health outcomes [18, 19], where psychological factors, such as beliefs and internal evaluative processes regarding one’s perceived weight, also have important consequences on health [20, 21]. Conversely, evidence of the degree of perceived weight relating to health adversity is inconsistent [22]. To the best of our knowledge, this study was the first to explore perceived weight, lifestyle risk, socio-demographic determinants and multimorbidity among young adult respondents. Our findings among a diverse, country-specific population with internet access show that socio-demographic determinants, weight perceptions, lifestyle risk, and multimorbidity varied significantly across the different economic countries. Higher morbidity and lifestyle risk scores were observed in those who reported an overweight weight perception when compared to those with an underweight or normal weight perception. In pooled analyses, the odds ratios in developing multimorbidity increased in those who perceived themselves as overweight, showing a larger odds ratio with high significance in those who reported 3 or more morbidities. Furthermore, this study showed that an overweight weight perception partially mediates the association between SEP and multimorbidity.

The multimorbidity challenge for LMICs such as South Africa and Kenya is ever-increasing. The burden of multimorbidity in LMICs is presently extensive [23], which may have resulted from rapid urbanisation, nutritional, and epidemiological transitions [24, 25] which are burdened on the already fragile healthcare systems in these settings [26, 27]. Across the countries, our survey findings show that cardiovascular morbidities, in particular, hypertension, myocardial infarction, and hypercholesterolemia/hyperlipidaemia were significantly higher in South Africa and Kenya (by > 2.2%) when compared to the UK, a HIC. Public health and healthcare system engagement in LMICs are therefore needed to adapt swiftly and effectively to overcome these challenges. While it is well documented that social determinants result in health inequalities [28], the unequal conditions in which people reside and work are dependent on several socio-demographic determinants such as sex, ethnicity, and SEP [29]. These tend to influence the distribution of risk factors that could potentially contribute to the development of morbid conditions, i.e., unhealthy dietary intake, physical inactivity, and deleterious lifestyle behaviours such as tobacco smoke and alcohol consumption are known risk factors for the development of hypertension [28]. To further highlight this trend, once diagnosed with hypertension, people with low SEP are less likely to afford antihypertensive medication, leading to uncontrolled hypertension and the early onset of further complications [28]. Lower socioeconomic groups, specifically seen in LMICs, are also more likely to consume unhealthy diets and frequent unhealthy lifestyle behaviours [30]. Although our survey results show that the SEP between South Africa and the UK was comparable, the SEP of the Kenyan respondents was substantially lower than that of the two aforementioned countries. Nevertheless, respondents from a LMIC (South Africa) consistently showed a higher percentage of respondents who smoke (36.9%) and consume alcohol (71.5%) when compared to those from a HIC (UK).

Presently, LMICs are facing a twofold burden of malnutrition, namely the coexistence of underweight and overweight/obesity [31]. With more than 650 million obese populations reported globally, nearly half of these populations reside in just 10 countries, 6 of which are low-to-middle income [28]. Despite the increased prevalence of obesity, weight concern, and recent weight control practices, obesity – irrespective of its detrimental aftereffects – is steadily increasing [32, 33]. It has been reported that weight control behaviours are triggered by body weight perceptions or the personal evaluation of one's weight irrespective of actual body mass index [33]. Individuals with an overweight weight perception are more likely to engage in weight reduction activities, whereas individuals who perceive themselves as normal weight but who have excess body weight will not involve themselves in weight loss behaviours [34, 35]. However, perceived weight is influenced by several factors being socio-demographic [36, 37], region specific or even a sociocultural component such as the social norms, beliefs, values, and expectations that arise about body size ideals and what is considered a normal body weight [38]. Overall, our survey results showed a great proportion of respondents from all three countries perceived themselves as normal weight [South Africa (64.8%), Kenya (74.8%), UK (54.1%)]. When compared to the proportion of those who perceived themselves as underweight, a greater proportion of respondents from all three countries considered themselves as overweight [South Africa (22.7%), Kenya (15.7%), UK (38.4%)]. Previous knowledge on those who perceive themselves as overweight are typically those individuals who most likely consume a healthier diet, engage in more physical activity and experience overall better health [39] in efforts to reduce excess weight. Contrary to the latter, results from our survey showed that those who perceived themselves as overweight were more physically inactive (44.7%) and displayed both a higher morbidity and lifestyle risk score when compared to those who perceived themselves as underweight or normal weight. This finding contradicts previous reports that weight control behaviours are precipitated by perceived weight [40]. Although this finding was unexpected when compared too previously reported findings in overweight perceived cohorts [39, 41], nearly 50% of those respondents who perceived themselves as overweight, had obesity as a confirmed clinic condition, rendering an explanation for this unexpected result. In addition, although both perceived weight and actual weight status influence self-rated health and life satisfaction, perceptions are more closely related to these outcomes [42]. Therefore, understanding the possible mechanisms through which perceived weight affects one’s health, will inevitably increase our overall understanding of the consequences surrounding weight and obesity.

With regards to the consequences of obesity, the most predominant result from our survey findings is the increased odds of multimorbidity in those who perceived themselves as overweight. Bearing in mind, nearly 50% of the respondents in the perceived overweight group, had obesity as a clinically diagnosed condition, these perceived overweight respondents were multiple times more likely to report multimorbidity consisting of 2 or more morbidities, which echoes previous research surrounding obesity and health outcomes [18, 19]. Socioeconomic position played an important role, while residing in Kenya – a LMIC country with numerous inequalities – also had significant contributions to our model. This significance of socio-economic contributors reflects previous research showing a more pronounced link between multimorbidity and perceived weight in those with a lower SEP [43, 44]. Stress is one potential mechanism [45] and poverty is a common source of stress [46], in conjunction with that the fact that being economically disadvantaged is taxing [47]. Bias in perceived weight increases the probability of suffering from medium or high psychological distress [48]. A 6-year prospective study examined the effects of poverty and psychosocial stress on central adiposity and found that individuals living in impoverished neighbourhoods who were also unfairly treated were at an increased risk of developing central adiposity [49]. There are, however, very few longitudinal studies that have explored the relationships between stress, body weight, and weight-related perceptions and behaviours. Though, it is plausible that obesity is a consequence of stress, as seen in reflecting the use of maladaptive coping strategies such as comfort eating or excessive sedentary behaviours [50]. This present study further extends the literature on SEP and multimorbidity as shown by the mediation analyses which suggested that weight perceptions, specifically having an overweight weight perception, had significant mediation effects on the relationship between SEP and multimorbidity. These mediation effects were highly significant and further added to our notion that SEP plays a significant contributing role to one’s overall health and that this relationship is mediated by perceived weight, i.e., being overweight. Research related to obesity reduction and prevention must understand the mediators and precursors of behaviour so that effective interventions can be developed.

This study was cross-sectional designed and therefore cannot infer causality. The absence of medical testing to confirm or newly diagnose multimorbidity was confirmed as a limitation, as well as the lack of acquiring the respondents' body mass index to accurately classify the respondents into their weight category, is subject to respondent bias. This study was also, in part, limited to a sample of relatively young adults with internet access, which is not representative of the entire youth or general population. Our results should, therefore, rather be interpreted in relation to the targeted respondents and not generalised for all young adults in this age category. There was a potential risk of sample independence being violated, however, quality control of the online platform ensured that only a single response was acquired from a single respondent. We would also like to acknowledge that SEP as measured by household asset score in our study is not the only measure of SEP and the use of this measure in relation to UK respondents may be less appropriate. Nevertheless, considering that our survey consisted of two LMICs (South Africa and Kenya), the use of household assets score was done so as a proxy of SEP. In addition, not only is the household assets score seen as the preferred measure of SEP in LMICs, it has also been shown to be central in the economic assessment of the household and is sensitive to change over time. Still, understanding multimorbidity and the perceived weight in younger individuals has the latent effect to decrease the risk of multimorbidity, especially in LMICs, via intervention strategies that are targeted at reducing risk factors (i.e., dietary intake, smoking, alcohol consumption, and physical inactivity). To substantiate the results we observed, studies with larger sample sizes are encouraged that also include respondents from other countries and a broader age range. It would also be particularly informative to assess the association between perceived weight and physical activity while considering country-specific barriers to physical activity as a potential mediator.

## Conclusions

To conclude, the results from our survey showed that SEP, perceived weight, lifestyle risk and multimorbidity varied significantly across the different economic countries. Those who perceived themselves as overweight reported a higher morbidity and lifestyle risk score when compared to those respondents who perceived themselves as underweight or normal weight. We also found that the odds of respondents who report having 2 or more morbidities increased multiple times with reporting an overweight weight perception. Having an overweight weight perception was also found to partially mediate the association between SEP and multimorbidity. The results from this study, therefore, confirm that those who perceive themselves as overweight had poorer health outcomes. This, consequently, highlights the significance of targeted intervention strategies aimed at improving weight-related awareness and potential risk factors.

## Supplementary Information

**Additional file 1: Supplementary Table S1.** General characteristics of young adults from multi-country survey.

**Additional file 2: Supplementary Table S2.** General characteristics of young adults from multi-country survey stratified by country and sex.

**Additional file 3: Supplementary Table S3.** Multivariable adjusted multinomial logistic regressions to determine the odds of having an adverse health profile with weight perceptions (pooled analysis).

## Data Availability

All data generated or analysed during this study are included in this published article [and its supplementary information files].

## Abbreviations

AIDS: Acquired immunodeficiency syndrome
ANOVA: Analyses of variance
CoE: Centre of Excellence
DSI-NRF: Department of Science and Innovation-National Research Foundation
GPAQ: Global physical activity questionnaire
gSEM: Generalised structural equation model
HCL/HDL: Hypercholesterolemia/hyperlipidemia
HIC: High-income country
HIV: Human immunodeficiency virus
HTN: Hypertension
IC: Information criteria
KE: Kenya
LMIC: Low-to-middle income country
MI: Myocardial infarction
MVPA: Moderate-to-vigorous physical activity
*n*: Number of participants
RSA: Republic of South Africa
SEP: Socioeconomic position
SD: Standard deviation
TB: Tuberculosis
UK: United Kingdom

## References

[1] Rossouw HA, Grant CC, Viljoen M (2012). Overweight and obesity in children and adolescents: The South African Problem. S Afr J Sci.

[2] den Engelsen C, Vos RC, Rijken M, Rutten GE (2015). Comparison of perceptions of obesity among adults with central obesity with and without additional cardiometabolic risk factors and among those who were formally obese, 3 years after screening for central obesity. BMC Public Health.

[3] van den Akker M, Buntinx F, Knottnerus JA (1996). Comorbidity or multimorbidity: what’s in a name? A review of literature. Eur J Gen Pract.

[4] Barnett K, Mercer SW, Norbury M (2012). Epidemiology of multimorbidity and implications for health care, research and medical education: a cross-sectional study. Lancet.

[5] Agborsangaya CB, Ngwakongnwi E, Lahtinen M, Cooke T, Johnson JA (2013). Multimorbidity prevalence in the general population: the role of obesity in chronic disease clustering. BMC Public Health.

[6] World Health Organization. Nutrition, Physical Activity and Obesity. https://www.euro.who.int/__data/assets/pdf_file/0020/243335/United-Kingdom-WHO-Country-Profile.pdf (2013). Accessed 25 Nov 2022.

[7] Kearney PM, Whelton M, Reynolds K, Muntner P, Whelton PK, He J (2005). Global burden of hypertension: Analysis of worldwide data. Lancet.

[8] Jiang SZ, Lu W, Zong XF, Ryan HY, Liu Y (2016). Obesity and hypertension. Exp Ther Med.

[9] Adeniyi OV, Yogeswaran P, Longo-Mbenza B, Goon DT (2016). Uncontrolled hypertension and its determinants in patients with concomitant type 2 diabetes mellitus (T2DM) in rural South Africa. PLoS ONE.

[10] The Academy of Medical Sciences. Improving the prevention and management of multimorbidity in sub-Saharan Africa. 2019. https://acmedsci.ac.uk/file-download/65601508#:~:text=Chronic%20infectious%20diseases%2C%20particularly%20HIV,to%20affect%20younger%20age%20groups. Accessed 23 Jan 2023.

[11] Neter JE, Stam BE, Kok FJ, Grobbee DE, Geleijnse JM (2003). Influence of weight reduction on blood pressure: a meta-analysis of randomized controlled trials. Hypertension.

[12] Wing RR, Goldstein MG, Acton KJ, Birch LL, Jakicic JM, Sallis JF, Smith-West D, Jeffery RW, Surwit RS (2001). Behavioral science research in diabetes: lifestyle changes related to obesity, eating behavior, and physical activity. Diabetes Care.

[13] Patte KA, Laxer RE, Qian W, Leatherdale ST (2016). An analysis of weight perception and physical activity and dietary behaviours among youth in the COMPASS study. SSM Popul Health.

[14] Mtintsilana A, Craig A, Mapanga W, Dlamini SN, Norris SA (2023). Association between socio-economic status and non-communicable disease risk in young adults from Kenya, South Africa, and the United Kingdom. Sci Rep.

[15] Curtice J. Attitudes to obesity: findings from the 2015 British social attitudes survey. Nat Cen Soc Res. 2015; 2015. https://www.bl.uk/collection-items/attitudes-to-obesity-findings-from-the-2015-british-social-attitudes-survey. Accessed 25 July 2022.

[16] Bull FC, Al-Ansari SS, Biddle S, Borodulin K, Buman MP, Cardon G, Carty C, Chaput JP, Chastin S, Chou R (2020). World Health Organization 2020 guidelines on physical activity and sedentary behaviour. Br J Sports Med.

[17] Global Physical Activity Questionnaire data cleaning. https://www.who.int/ncds/surveillance/steps/resources/GPAQ_Analysis_Guide.pdf?ua=1. Accessed 11 Aug 2022.

[18] Williams J, Wake M, Hesketh K, Maher E, Waters E (2005). Health-related quality of life of overweight and obese children. JAMA-J AM MED ASSOC.

[19] World Health Organisation. Obesity. 2022. https://www.who.int/health-topics/obesity. Accessed 25 Nov 2022.

[20] McCabe MP, Ricciardelli LA, Sitaram G, Mikhali K (2006). Accuracy of body size estimation: Role of biopsychological variables. Body Image.

[21] Gillen MM (2015). Associations between positive body image and indicators of men’s and women’s mental and physical health. Body Image.

[22] Darimont T, Karavasiloglou N, Hysaj O, Richard A, Rohrmann S (2020). Body weight and self-perception are associated with depression: results from the National Health and Nutrition Examination Survey (NHANES) 2005–2016. J Affect Disord.

[23] WHO. NCD Global monitoring framework. 2011. https://www.who.int/nmh/global_monitoring_framework/en/. Accessed 10 Dec 2022.

[24] Miranda JJ, Barrientos-Gutierres T, Corvalan C, Hyder AA, Lazo-Porras M, Oni T, Wells JCK (2019). Understanding the rise of cardiometabolic diseases in low- and middle-income countries. Nat Med.

[25] Mendoza W, Miranda JJ (2017). Global shifts in cardiovascular disease, the epidemiologic transition and other contributing factors: toward a new practice of global health cardiology. Cardiol Cin.

[26] Muka T, Imo D, Jaspers L, Colpani V, Chaker L, van der Lee SJ (2015). The global impact of non-communicable diseases on healthcare spending and national income: a systematic review. Eur J Epidemiol.

[27] Frenk J, Bobaclilla JL, Sepulveda J, Cervantes ML (1989). Health transition in middle-income countries: new challenges for health care. Health Policy Plan.

[28] Schutte AE, Srinivasapura Venkateshmurthy N, Mohan S, Prabhakaran D (2021). Hypertension in Low- and Middle-Income Countries. Circ Res.

[29] Marmot M, Friel S, Bell R, Houweling TA, Taylor S (2008). Commission on Social Determinants of Health. Closing the gap in a generation: health equity through action on the social determinants of health. Lancet..

[30] Allen L, Williams J, Townsend N, Mikkelsen B, Roberts N, Foster C, Wickramasinghe K (2017). Socioeconomic status and non-communicable disease behavioural risk factors in low-income and lower-middle-income countries: a systematic review. Lancet Glob Health.

[31] Popkin BM, Corvalan C, Grummer-Strawn LM (2020). Dynamics of the double burden of malnutrition and the changing nutrition reality. Lancet.

[32] Lowry R, Galuska D, Fulton JE (2002). Weight management goals and practices among US high school students: associations with physical activity, diet, and smoking. J of Adolescent Health..

[33] Irving LM, Wall M, Neuwark-Sztainer D, Story M (2001). Steroid use among adolescents: findings from project EAT. J Adolesc Health.

[34] Wong MMC (2010). Body weight perception and methods of weight reduction used by patients with first-episode psychotic disorders in Hong Kong. East Asian Arch Psychiatry.

[35] Brener ND, Eaton DK, Lowry R, McManus T (2004). The association between weight perception and BMI among high school students. Obes Res.

[36] Gregory CO, Blank HM, Gillespie C, Maynard LM, Serdula MK (2008). Health perceptions and demographic characteristics associated with underassessment of body weight. Obesity.

[37] Kim KHC (2007). Religion weight perception, and weight control behavior. Eating Behaviors..

[38] Wardle J, Waller J, Rapoport L (2001). Body dissatisfaction and binge eating in obese women: The role of restraint and depression. Obes Res.

[39] Edwards NM, Pettingell S, Borowsky IW (2010). Where perception meets reality: self-perception of weight in overweight adolescents. Pediatrics.

[40] Wang Y, Liang H, Chen X. Measured body mass index, body weight perception, dissatisfaction and control practices in urban, low-income African American adolescents. BMC Public Health. 2009;9:183. 10.1186/1471-2458-9-183 PMC2704208 19523206

[41] Duncan DT, Wolin KY, Scharoun-Lee M, Ding EL, Warner ET, Bennett GG. Does perception equal reality? Weight misperception in relation to weight-related attitudes and behaviors among overweight and obese US adults. Int J Behav Nutr Phys Act. 2011;8:20. 10.1186/1479-5868-8-20 PMC3073863 21426567

[42] Herman KM, Hopman WM, Rosenberg MW (2013). Self-rated health and life satisfaction among Canadian adults: associations of perceived weight status vs BMI. Qual Life Res.

[43] Ball K, Crawford D (2005). Socioeconomic status and weight change in adults: a review. Soc Sci Med.

[44] McLaren L (2007). Socioeconomic status and obesity. Epidemiol Rev.

[45] Mackenbach JP. Health Inequalities: Europe in Profile. Rotterdam: Erasmus MC;2006. http://www.who.int/social_determinants/resources.

[46] American Psychological Association (APA) (2015). Stress in America: Paying with Our Health.

[47] Mani A, Mullainathan S, Shafir E, Zhao J (2013). Poverty impedes cognitive function. Science.

[48] Atlantis E, Ball K (2008). Association between weight perception and psychological distress. Int J Obes.

[49] Kwarteng JL, Schulz AJ, Mentz GB, Israel BA, Perkins DW (2017). Independent Effects of Neighborhood Poverty and Psychosocial Stress on Obesity Over Time. J Urban Health.

[50] Sulkowski ML, Dempsey J, Dempsey AG (2011). Effects of stress and coping on binge eating in female college students. Eat Behav.

