# Supplementary material for: Socioeconomic position, perceived weight, lifestyle risk, and multimorbidity in young adults aged 18 to 35 years: a Multi-country Study
Source: BMC Public Health. 2023 Jul 15;23:1360. doi: 10.1186/s12889-023-16234-1 (PMC10349474; doi:10.1186/s12889-023-16234-1)
Supplement: Supplementary file 1 — Additional file 1: Supplementary Table S1. General characteristics of young adults from multi-country survey. [file 12889_2023_16234_MOESM1_ESM.docx]

**Supplementary Table S1. General characteristics of young adults from multi-country survey.**

|  |  | **UK**  (*n*=1000) | **South Africa**  (*n*=1000) | **Kenya**  (*n*=1000) | ***p-*trend** |
| --- | --- | --- | --- | --- | --- |
| **Socioeconomic position** | | | | | |
| Household assets score | (mean ± SD) | 14.4 ± 4.24 **^c^** | 14.5 ± 4.27 ^b^ | 11.6 ± 4.51 ^c^ | **<0.001** |
| **Health information** | | | | | |
| Number of morbidities | (mean ± SD) | 1.26 ± 0.83 | 1.34 ± 1.21 | 1.33 ± 1.03 | 0.19 |
| 1 morbidity | *n* (%) | 849 (84.9%) | 837 (83.7%) | 826 (82.6%) | 0.67 |
| 2 morbidities | *n* (%) | 95 (9.5%) | 99 (9.9%) | 104 (10.4%) |  |
| 3+ morbidities | *n* (%) | 55 (5.5%) | 57 (5.7%) | 68 (6.8%) |  |
| Hypertension (yes) | *n* (%) | 100 (10.0%) **^ac^** | 156 (15.6%) **^a^** | 156 (15.6%) **^c^** | **<0.001** |
| Myocardial infarction (yes) | *n* (%) | 16 (1.6%) **^ac^** | 63 (6.3%) **^a^** | 62 (6.2%) **^c^** | **<0.001** |
| Stroke (yes) | *n* (%) | 20 (2.0%) | 14 (1.4%) | 13 (1.3%) | 0.40 |
| Hypercholesterolemia / hyperlipidaemia (yes) | *n* (%) | 72 (7.2%) **^a^** | 117 (11.7%) **^a^** | 94 (9.4%) | **0.003** |
| Diabetes (yes) | *n* (%) | 73 (7.3%) | 77 (7.7%) | 78 (7.8%) | 0.91 |
| Obesity (yes) | *n* (%) | 225 (22.5%) **^a^** | 164 (16.4%) **^a^** | 204 (20.4%) | **0.002** |
| HIV/AIDS (yes) | *n* (%) | 10 (1.0%) **^a^** | 68 (6.8%) **^ab^** | 23 (2.3%) **^b^** | **<0.001** |
| Tuberculosis (yes) | *n* (%) | 14 (1.4%) **^ac^** | 50 (5.0%) **^ab^** | 85 (8.5%) **^bc^** | **<0.001** |
| Asthma/lung disease (yes) | *n* (%) | 162 (16.2%) **^c^** | 133 (13.3%) | 111 (11.1%) **^c^** | **0.004** |
| Cancer (yes) | *n* (%) | 15 (1.5%) | 16 (1.6%) | 13 (1.3%) | 0.85 |
| Liver disease (yes) | *n* (%) | 12 (1.2%) | 14 (1.4%) | 18 (1.8%) | 0.52 |
| Chronic kidney disease (yes) | *n* (%) | 13 (1.3%) | 17 (1.7%) | 14 (1.4%) | 0.74 |
| Mental health risk (anxiety/depression/bi-polar) (yes) | *n* (%) | 378 (37.8%) **^ac^** | 267 (26.7%) **^a^** | 281 (28.1%) **^c^** | **<0.001** |
| Joint disease (arthritis) (yes) | *n* (%) | 149 (14.9%) | 179 (17.9%) | 177 (17.7%) | 0.13 |
| **Lifestyle risk information** | | | | | |
| Smoke (yes) | *n* (%) | 296 (29.6%) **^ac^** | 369 (36.9%) **^ab^** | 170 (17.0%) **^bc^** | **<0.001** |
| Alcohol (yes) | *n* (%) | 627 (62.7%) **^ac^** | 715 (71.5%) **^ab^** | 509 (50.9%) **^bc^** | **<0.001** |
| Vigorous exercise |  |  |  |  |  |
| 0-1 days/week | *n* (%) | 375 (53.4%) **^ac^** | 241 (34.3%) **^ab^** | 86 (12.3%) **^bc^** | **<0.001** |
| 2-4 days/week | *n* (%) | 443 (32.0%) | 544 (39.3%) | 396 (28.6%) |  |
| 5-7 days/week | *n* (%) | 182 (19.9%) | 215 (23.5%) | 518 (56.6%) |  |
| Moderate exercise |  |  |  |  |  |
| 0-1 days/week | *n* (%) | 367 (47.8%) **^c^** | 310 (40.4%) **^b^** | 90 (11.7%) **^bc^** | **<0.001** |
| 2-4 days/week | *n* (%) | 405 (30.5%) | 494 (37.3%) | 427 (32.2%) |  |
| 5-7 days/week | *n* (%) | 288 (25.1%) | 196 (21.6%) | 483 (53.3%) |  |
| Walking |  |  |  |  |  |
| 0-3 days/week | *n* (%) | 308 (27.1%) **^c^** | 292 (25.7%) **^b^** | 536 (47.2%) **^bc^** | **<0.001** |
| 4-6 days/week | *n* (%) | 301 (36.0%) | 348 (41.6%) | 187 (22.4%) |  |
| 7 days/week | *n* (%) | 391 (38.0%) | 360 (35.0%) | 277 (26.9%) |  |
| Physical inactivity (MVPA<60mins/day) | *n* (%) | 95 (35.8%) | 439 (27.3%) | 285 (44.7%) | **<0.001** |
| Lifestyle risk score | (mean ± SD) | 1.59 ± 0.947 **^c^** | 1.55 ± 0.926 ^b^ | 1.13 ± 0.922 ^c^ | **<0.001** |
| **Weight perceptions** | | | | | |
| Perceived underweight status | *n* (%) | 61/817 (7.5%) | 103/820 (12.6%) | 101/873 (11.6%) | **0.002** |
| Perceived normal weight status | *n* (%) | 442/817 (54.1%) **^ac^** | 531/820 (64.8%) **^ab^** | 635/873 (74.8%) **^bc^** | **<0.001** |
| Perceived overweight status | *n* (%) | 314/817 (38.4%) **^ac^** | 186/820 (22.7%) **^a^** | 137/873 (15.7%) **^c^** | **<0.001** |
| Perceptions of being overweight | | | | | |
| It is inherited (yes) | *n* (%) | 287 (28.7%) **^ac^** | 196 (19.6%) **^a^** | 164 (16.4%) **^c^** | **<0.001** |
| Due to a slow metabolism (yes) | *n* (%) | 348 (34.8%) **^ac^** | 474 (47.4%) **^a^** | 488 (48.8%) **^c^** | **<0.001** |
| Overindulge (yes) | *n* (%) | 633 (63.3%) **^c^** | 585 (58.5%) **^b^** | 367 (36.7%) **^bc^** | **<0.001** |
| Physically inactive (yes) | *n* (%) | 673 (67.3%) **^ac^** | 568 (56.8%) **^a^** | 558 (55.8%) **^c^** | **<0.001** |

Abbreviations: *n* – number of participants; UK – United Kingdom. Bold values denote statistical significance (p<0.05). **^a^** Significant difference between Kenya and South Africa; **^b^** significant difference between South Africa and UK; **^c^** significant difference between Kenya and UK.
