# Supplementary material for: Socioeconomic position, perceived weight, lifestyle risk, and multimorbidity in young adults aged 18 to 35 years: a Multi-country Study
Source: BMC Public Health. 2023 Jul 15;23:1360. doi: 10.1186/s12889-023-16234-1 (PMC10349474; doi:10.1186/s12889-023-16234-1)
Supplement: Supplementary file 2 — Additional file 2: Supplementary Table S2. General characteristics of young adults from multi-country survey stratified by country and sex. [file 12889_2023_16234_MOESM2_ESM.docx]

**Supplementary Table S2. General characteristics of young adults from multi-country survey stratified by country and sex.**

|  |  | **UK**  (*n*=1000) | | | **South Africa**  (*n*=1000) | | | **Kenya**  (*n*=1000) | | |
| --- | --- | --- | --- | --- | --- | --- | --- | --- | --- | --- |
|  |  | **Men**  (*n*=500) | **Women**  (*n*=500) | ***p* value** | **Men**  (*n*=500) | **Women**  (*n*=500) | ***p* value** | **Men**  (*n*=500) | **Women**  (*n*=500) | ***p* value** |
| **Socioeconomic position** | | | | | | | | | | |
| Household assets score | (mean ± SD) | 14.3 ± 4.89 | 14.5 ± 3.48 | 0.40 | 14.1 ± 4.80 | 14.9 ± 3.63 | **0.004** | 11.4 ± 4.84 | 11.8 ± 4.15 | 0.20 |
| **Health information** | | | | | | | | | | |
| Number of morbidities | (mean ± SD) | 1.23 ± 0.862 | 1.29 ± 0.801 | 0.35 | 1.33 ± 1.29 | 1.34 ± 1.14 | 0.86 | 1.34 ± 1.06 | 1.31 ± 0.991 | 0.64 |
| 1 morbidity | *n* (%) | 434 (86.8%) | 415 (83.0%) | 0.21 | 424 (84.8%) | 413 (82.6%) | 0.53 | 406 (81.2%) | 420 (84.0%) | 0.10 |
| 2 morbidities | *n* (%) | 42 (8.4%) | 53 (10.6%) |  | 47 (9.4%) | 52 (10.4%) |  | 62 (12.4%) | 42 (8.4%) |  |
| 3+ morbidities | *n* (%) | 23 (4.6%) | 32 (6.4%) |  | 25 (5.0%) | 32 (6.4%) |  | 31 (6.2%) | 37 (7.4%) |  |
| Hypertension (yes) | *n* (%) | 52 (10.4%) | 48 (9.6%) | 0.67 | 74 (14.8%) | 82 (16.4%) | 0.49 | 66 (13.2%) | 90 (18.0%) | **0.036** |
| Myocardial infarction (yes) | *n* (%) | 9 (1.8%) | 7 (1.4%) | 0.61 | 35 (7.0%) | 28 (5.6%) | 0.36 | 34 (6.8%) | 28 (5.6%) | 0.43 |
| Stroke (yes) | *n* (%) | 14 (2.8%) | 6 (1.2%) | 0.071 | 9 (1.8%) | 5 (1.0%) | 0.28 | 7 (1.4%) | 6 (1.2%) | 0.78 |
| Hypercholesterolemia / hyperlipidaemia (yes) | *n* (%) | 35 (7.0%) | 37 (7.4%) | 0.81 | 68 (13.6%) | 49 (9.8%) | 0.062 | 55 (11.0%) | 39 (7.8%) | 0.083 |
| Diabetes (yes) | *n* (%) | 42 (8.4%) | 31 (6.2%) | 0.18 | 37 (7.4%) | 40 (8.0%) | 0.72 | 47 (9.4%) | 31 (6.2%) | 0.059 |
| Obesity (yes) | *n* (%) | 104 (20.8%) | 121 (24.2%) | 0.20 | 68 (13.6%) | 96 (19.2%) | **0.017** | 91 (18.2%) | 113 (22.6%) | 0.084 |
| HIV/AIDS (yes) | *n* (%) | 7 (1.4%) | 3 (0.6%) | 0.20 | 30 (6.0%) | 38 (7.6%) | 0.31 | 13 (2.6%) | 10 (2.0%) | 0.53 |
| Tuberculosis (yes) | *n* (%) | 7 (1.4%) | 7 (1.4%) | 1.00 | 30 (6.0%) | 20 (4.0%) | 0.15 | 45 (9.0%) | 40 (8.0%) | 0.57 |
| Asthma (yes) | *n* (%) | 76 (15.2%) | 86 (17.2%) | 0.39 | 68 (13.6%) | 65 (13.0%) | 0.78 | 58 (11.6%) | 53 (10.6%) | 0.62 |
| Cancer (yes) | *n* (%) | 10 (2.0%) | 5 (1.0%) | 0.19 | 9 (1.8%) | 7 (1.4%) | 0.62 | 6 (1.2%) | 7 (1.4%) | 0.78 |
| Liver disease (yes) | *n* (%) | 6 (1.2%) | 6 (1.2%) | 1.00 | 6 (1.2%) | 8 (1.6%) | 0.59 | 11 (2.2%) | 7 (1.4%) | 0.34 |
| Chronic kidney disease (yes) | *n* (%) | 7 (1.4%) | 6 (1.2%) | 0.78 | 11 (2.2%) | 6 (1.2%) | 0.22 | 9 (1.8%) | 5 (1.0%) | 0.28 |
| Mental health risk (yes) | *n* (%) | 177 (35.4%) | 201 (40.2%) | 0.12 | 119 (23.8%) | 148 (29.6%) | **0.038** | 137 (27.4%) | 144 (28.8%) | 0.62 |
| Joint disease (arthritis) (yes) | *n* (%) | 70 (14.0%) | 79 (15.8%) | 0.42 | 100 (20.0%) | 79 (15.8%) | 0.083 | 93 (18.6%) | 84 (16.8%) | 0.46 |
| **Lifestyle related information** | | | | | | | | | | |
| Smoke (yes) | *n* (%) | 103 (38.6%) | 247 (20.6%) | **<0.001** | 247 (49.4%) | 122 (24.4%) | **<0.001** | 116 (23.2%) | 54 (10.8%) | **<0.001** |
| Alcohol (yes) | *n* (%) | 344 (68.8%) | 283 (56.6%) | **<0.001** | 376 (75.2%) | 339 (67.8%) | **0.006** | 279 (55.8%) | 230 (46.0%) | **0.001** |
| Vigorous exercise |  |  |  |  |  |  |  |  |  |  |
| 0-1 days/week | *n* (%) | 154 (30.8%) | 221 (44.2%) | **<0.001** | 90 (18.0%) | 151 (30.2%) | **<0.001** | 26 (5.2%) | 60 (12.0%) | **<0.001** |
| 2-4 days/week | *n* (%) | 230 (46.0%) | 213 (42.6%) |  | 286 (57.2%) | 258 (51.6%) |  | 196 (39.2%) | 200 (40.0%) |  |
| 5-7 days/week | *n* (%) | 116 (23.2%) | 66 (13.2%) |  | 124 (24.8%) | 91 (18.2%) |  | 278 (55.6%) | 240 (48.0%) |  |
| Moderate exercise |  |  |  |  |  |  |  |  |  |  |
| 0-1 days/week | *n* (%) | 150 (30.0%) | 217 (43.4%) | **<0.001** | 125 (25.0%) | 185 (37.0%) | **<0.001** | 33 (6.6%) | 57 (11.4%) | **0.006** |
| 2-4 days/week | *n* (%) | 215 (43.0%) | 190 (38.0%) |  | 265 (53.0%) | 229 (45.8%) |  | 206 (41.2%) | 221 (44.2%) |  |
| 5-7 days/week | *n* (%) | 135 (27.0%) | 93 (18.6%) |  | 110 (22.0%) | 86 (17.2%) |  | 261 (52.2%) | 222 (44.4%) |  |
| Walking |  |  |  |  |  |  |  |  |  |  |
| 0-3 days/week | *n* (%) | 155 (31.0%) | 153 (30.6%) | 0.46 | 122 (24.4%) | 170 (34.0%) | **0.003** | 281 (56.2%) | 255 (51.0%) | 0.10 |
| 4-6 days/week | *n* (%) | 142 (28.4%) | 159 (31.8%) |  | 190 (38.0%) | 158 (31.6%) |  | 81 (16.2%) | 106 (21.2%) |  |
| 7 days/week | *n* (%) | 203 (40.6%) | 188 (37.6%) |  | 188 (37.6%) | 172 (34.4%) |  | 138 (27.6%) | 139 (27.8%) |  |
| Lifestyle risk score | (mean ± SD) | 1.66 ± 0.944 | 1.52 ± 0.946 | **0.021** | 1.63 ± 0.880 | 1.48 ± 0.965 | **0.008** | 1.15 ± 0.946 | 1.10 ± 0.898 | 0.36 |
| **Weight perceptions** | | | | | | | | | | |
| Perceived underweight status | *n* (%) | 43/389 (11.1%) | 18/429 (4.2%) | **<0.001** | 59/393 (15.0%) | 44/427 (10.3%) | **0.042** | 46/435 (10.6%) | 55/438 (12.6%) | 0.36 |
| Perceived normal weight status | *n* (%) | 215/389 (55.3%) | 227/429 (52.9%) | 0.52 | 281/393 (71.5%) | 250/427 (58.5%) | **<0.001** | 352/435 (80.9%) | 283/438 (64.6%) | **<0.001** |
| Perceived overweight status | *n* (%) | 131/389 (33.7%) | 183/429 (42.7%) | **0.008** | 53/393 (13.5%) | 133/427 (31.1%) | **<0.001** | 37/435 (8.5%) | 100/438 (22.8%) | **<0.001** |
| Perceptions of being overweight | | | | | | | | | | |
| It is inherited (yes) | *n* (%) | 173 (34.6%) | 114 (22.8%) | **<0.001** | 113 (22.6%) | 83 (16.6%) | **0.017** | 88 (17.6%) | 76 (15.2%) | 0.31 |
| Due to a slow metabolism (yes) | *n* (%) | 198 (39.6%) | 150 (30.0%) | **0.001** | 235 (47.0%) | 239 (47.8%) | 0.80 | 237 (47.4%) | 251 (50.2%) | 0.38 |
| Overindulge (yes) | *n* (%) | 325 (65.0%) | 308 (61.6%0 | 0.27 | 320 (64.0%) | 265 (53.0%) | **<0.001** | 191 (38.2%) | 176 (35.2%) | 0.33 |
| Physically inactive (yes) | *n* (%) | 349 (69.8%) | 324 (64.8%) | 0.092 | 309 (61.8%) | 259 (51.8%) | **0.001** | 306 (61.2%) | 252 (50.4%) | **<0.001** |

Bold values denote statistical significance (p<0.05).
