# Supplementary material for: Socioeconomic position, perceived weight, lifestyle risk, and multimorbidity in young adults aged 18 to 35 years: a Multi-country Study
Source: BMC Public Health. 2023 Jul 15;23:1360. doi: 10.1186/s12889-023-16234-1 (PMC10349474; doi:10.1186/s12889-023-16234-1)
Supplement: Supplementary file 3 — Additional file 3: Supplementary Table S3. Multivariable adjusted multinomial logistic regressions to determine the odds of having an adverse health profile with weight perceptions (pooled analysis). [file 12889_2023_16234_MOESM3_ESM.docx]

**Supplementary Table S3. Multivariable adjusted multinomial logistic regressions to determine the odds of having an adverse health profile with weight perceptions (pooled analysis).**

|  |  |  | **Health profile** | | | | | | | | |
| --- | --- | --- | --- | --- | --- | --- | --- | --- | --- | --- | --- |
|  |  |  | **0-1 Morbidities**  (*n*= 2512) | | | **2 Morbidities**  (*n*= 298) | | | **≥3 Morbidities**  (*n*= 180) | | |
|  |  |  | **OR** | **β (95 % Cl)** | ***p* value** | **OR** | **β (95 % Cl)** | ***p* value** | **OR** | **β (95 % Cl)** | ***p* value** |
| **Model 1** | **Weight perception** | Underweight | (reference) | | | 1.083 | 0.079 (0.681; 1.723) | 0.74 | 1.789 | 0.582 (1.009; 3.173) | **0.047** |
|  |  | Normal weight |  |  |  |  | (reference) |  |  | (reference) |  |
|  |  | Overweight |  |  |  | 2.241 | 0.807 (1.693; 2.966) | **<0.001** | 3.656 | 1.296 (2.528; 5.286) | **<0.001** |
| **Model 2** | **Weight perception** | Underweight |  |  |  | 1.085 | 0.082 (0.680; 1.733) | 0.73 | 1.819 | 0.599 (1.021; 3.242) | **0.042** |
|  |  | Normal weight |  |  |  |  | (reference) |  |  | (reference) |  |
|  |  | Overweight |  |  |  | 2.426 | 0.886 (1.803; 3.265) | **<0.001** | 4.037 | 1.396 (2.732; 5.967) | **<0.001** |
|  | Country | UK |  |  |  |  | (reference) |  |  | (reference) |  |
|  |  | South Africa |  |  |  | 1.340 | 0.293 (0.956; 1.880) | 0.089 | 1.278 | 0.245 (0.806; 2.026) | 0.30 |
|  |  | Kenya |  |  |  | 1.552 | 0.439 (1.104; 2.181) | **0.011** | 1.981 | 0.684 (1.274; 3.081) | **0.002** |
|  | Age category | 18-21yrs |  |  |  |  | (reference) |  |  | (reference) |  |
|  |  | 22-25yrs |  |  |  | 1.880 | 0.631 (1.155; 3.061) | **0.011** | 1.439 | 0.364 (0.762; 2.720) | 0.26 |
|  |  | 26-29yrs |  |  |  | 1.324 | 0.281 (0.795; 2.203) | 0.28 | 1.621 | 0.483 (0.866; 3.035) | 0.13 |
|  |  | 30-35yrs |  |  |  | 2.011 | 0.698 (1.243; 3.252) | **0.004** | 1.487 | 0.397 (0.794; 2.786) | 0.22 |
|  | Sex | Male |  |  |  |  | (reference) |  |  | (reference) |  |
|  |  | Female |  |  |  | 0.917 | –0.087 (0.701; 1.199) | 0.53 | 1.135 | 0.127 (0.792; 1.626) | 0.49 |
| **Model 3** | **Weight perception** | Underweight |  |  |  | 1.082 | 0.079 (0.677; 1.731) | 0.74 | 1.817 | 0.597 (1.014; 3.257) | **0.045** |
|  |  | Normal weight |  |  |  |  | (reference) |  |  | (reference) |  |
|  |  | Overweight |  |  |  | 2.330 | 0.846 (1.731; 3.136) | **<0.001** | 3.737 | 1.318 (2.528; 5.525) | **<0.001** |
|  | Country | UK |  |  |  |  | (reference) |  |  | (reference) |  |
|  |  | South Africa |  |  |  | 1.328 | 0.284 (0.947; 1.863) | 0.10 | 1.226 | 0.203 (0.772; 1.946) | 0.39 |
|  |  | Kenya |  |  |  | 1.893 | 0.638 (1.327; 2.701) | **<0.001** | 2.800 | 1.030 (1.771; 4.428) | **<0.001** |
|  | Age category | 18-21yrs |  |  |  |  | (reference) |  |  | (reference) |  |
|  |  | 22-25yrs |  |  |  | 1.834 | 0.606 (1.124; 2.990) | **0.015** | 1.375 | 0.319 (0.724; 2.614) | 0.33 |
|  |  | 26-29yrs |  |  |  | 1.270 | 0.239 (0.762; 2.117) | 0.36 | 1.513 | 0.414 (0.804; 2.848) | 0.20 |
|  |  | 30-35yrs |  |  |  | 1.904 | 0.644 (1.176; 3.083) | **0.009** | 1.389 | 0.328 (0.739; 2.609) | 0.31 |
|  | Sex | Male |  |  |  |  | (reference) |  |  | (reference) |  |
|  |  | Female |  |  |  | 0.926 | –0.077 (0.708; 1.211) | 0.57 | 1.194 | 0.177 (0.831; 1.716) | 0.34 |
|  | SEP score | Score |  |  |  | 1.072 | 0.069 (1.033; 1.112) | **<0.001** | 1.153 | 0.142 (1.094; 1.215) | **<0.001** |

Abbreviations: *n* – number of participants; SEP – socioeconomic position. Bold values denote statistical significance (p<0.05).
